# Sexual Health, Quality of Life, and Fertility Counselling in Breast Cancer Survivors Younger than 40 Years

**DOI:** 10.3390/medicina62061051

**Published:** 2026-05-28

**Authors:** Cristina Tanase-Damian, Nicoleta Zenovia Antone, Diana Loreta Paun, Ioan Tanase, Patriciu Achimaș-Cadariu

**Affiliations:** 1 Department of Medicine, Iuliu Hațieganu University of Medicine and Pharmacy, 400347 Cluj-Napoca, Romania; 2 Department of Oncology, Iuliu Hațieganu University of Medicine and Pharmacy, 400347 Cluj-Napoca, Romania; nicoleta.antone@iocn.ro; 3 Department of Oncology, The Oncology Institute “Prof. Dr. Ion Chiricuta”, 400015 Cluj-Napoca, Romania; 4 Department of Endocrinology, Carol Davila University of Medicine and Pharmacy, 050474 Bucharest, Romania; diana.paun@umfcd.ro; 5 Department of Surgery, Carol Davila University of Medicine and Pharmacy, 050474 Bucharest, Romania; ioan.tanase@umfcd.ro; 6 Department of Surgery, Iuliu Hațieganu University of Medicine and Pharmacy, 400347 Cluj-Napoca, Romania; pachimas@umfcluj.ro; 7 Department of Surgery, The Oncology Institute “Prof. Dr. Ion Chiricuta”, 400015 Cluj-Napoca, Romania

**Keywords:** young breast cancer survivors, sexual health, quality of life, fertility preservation, endocrine therapy, EORTC QLQ-C30, EORTC SHQ-22, oncofertility

## Abstract

*Background and Objectives*: Young women treated for breast cancer often face persistent sexual and reproductive challenges post-treatment, which frequently remain unaddressed during routine follow-up. This study evaluated health-related quality of life (HRQoL), sexual health, and fertility-related counselling in breast cancer survivors younger than 40 years. *Materials and Methods*: We performed a single-centre cross-sectional study including 65 women with non-metastatic breast cancer (at least 12 months post-primary treatment). Patients completed the EORTC QLQ-C30 and SHQ-22 questionnaires, along with a pilot-tested 7-item fertility preservation survey. Data were analyzed using descriptive statistics and non-parametric tests to compare treatment subgroups (e.g., endocrine therapy vs. others). *Results*: Overall quality-of-life scores were relatively preserved (median 66.67), with high functional scores. However, patients reported symptomatic distress, particularly fatigue and insomnia (both median 33.33). Sexual health was significantly impacted: communication with healthcare professionals was the most impaired domain (median score 100), followed by low libido (66.7) and vaginal dryness (66.7). Women receiving endocrine therapy reported significantly lower functional scores and a higher symptom burden compared to those not on endocrine treatment (*p* < 0.05). While 60% of patients received fertility information, only 16.9% underwent preservation procedures. Notably, a significant association was observed between reproductive health actions and sexual behaviour; women who had undergone fertility preservation procedures reported higher levels of sexual activity (*p* = 0.015). *Conclusions*: Despite relatively preserved functional status, young breast cancer survivors face significant symptomatic and sexual challenges. The profound lack of communication regarding sexual health and the gaps in fertility counselling highlight the need for a more integrative, multidisciplinary approach in survivorship care.

## 1. Introduction

Breast cancer remains the most frequently diagnosed malignancy among women worldwide, characterized by profound physical, emotional, and practical consequences. With an estimated 2.3 million new diagnoses each year, it continues to contribute significantly to cancer-specific mortality, with a reported fatality rate of approximately 7% in premenopausal adults [1,2]. The improvements in screening, systemic therapies, and multidisciplinary management have led to increased survival rates, resulting in a growing population of long-term survivors [1,2,3]. As a result, breast cancer care now extends beyond disease control and includes survivorship issues such as quality of life, psychosocial well-being, sexual health, and reproductive outcomes.

Sexual health is an important component of global health and can be affected by an oncologic diagnosis [4]. Breast cancer treatment—including surgery, chemotherapy, radiotherapy, endocrine therapy, and targeted agents—can negatively impact sexual function through mechanisms such as ovarian insufficiency, hormonal disruption, genitourinary syndrome of menopause, altered body image, neuropathy, fatigue, and psychological distress [5,6,7]. Previous studies indicate that 40–60% of breast cancer survivors experience persistent sexual dysfunction, including low desire, dyspareunia, arousal and orgasmic difficulties, and reduced sexual satisfaction [8]. These problems may negatively affect quality of life and are associated with anxiety, depression, and reduced adherence to therapy [4,9]. Despite these issues, sexual health continues to receive limited attention within routine oncologic care. Current evidence indicates that only a minority of patients experiencing sexual difficulties engage with or receive support from healthcare professionals [5,10,11,12].

For premenopausal patients, sexual dysfunction frequently coexists with fertility concerns. Chemotherapy-induced gonadotoxicity and prolonged endocrine therapy lead to diminished ovarian reserve and infertility [13,14]. Oncofertility has introduced strategies for fertility preservation, such as oocyte and embryo cryopreservation, ovarian tissue freezing, and ovarian suppression with gonadotropin-releasing hormone agonists during chemotherapy [15,16,17]. International guidelines from the American Society of Clinical Oncology (ASCO) and the European Society for Medical Oncology (ESMO) recommend fertility counselling for all reproductive-age patients prior to initiating cancer therapy [17,18]. Despite this, fertility preservation discussions remain inconsistently implemented in clinical practice, and many young survivors report difficulties related to reproductive planning and sexual well-being [17,19].

Genetic predispositions, such as germline BRCA1/2 pathogenic variants, add further complexity to survivorship care. Patient with BRCA mutations often undergo more aggressive surgeries and systemic treatment. They might also have risk-reducing salpingo-oophorectomy at a younger age, which can affect their ability to have children and their sexual health [20,21]. Moreover, the increasing application of assisted reproductive technologies (ART) among breast cancer survivors has raised clinical questions regarding ovarian stimulation outcomes, timing relative to systemic therapy, and long-term reproductive success rates [22,23,24].

To evaluate the multidimensional impact of breast cancer treatment on QoL, validated patient-reported outcome measures are essential. The European Organisation for Research and Treatment of Cancer (EORTC) developed instruments such as the QLQ-C30 and breast cancer-specific modules that assess global QoL, functional domains, symptom burden, and treatment side effects [25,26]. These tools enable systematic quantification of factors including fatigue, pain, body image concerns, sexual problems, and overall functioning, thereby capturing both physical and psychosocial sequelae of therapy.

In light of the growing cohort of young survivors and the importance of sexual and reproductive health in long-term QoL, a cross-disciplinary integration of oncology, reproductive medicine, psycho-oncology, and survivorship care is needed.

## 2. Materials and Methods

### 2.1. Study Design and Subject Recruitment

This unicentric cross-sectional study was conducted at the Cancer Institute “Prof. Ion Chiricuta”, Cluj-Napoca, Romania. The Institute’s internal review board approved the study protocol. Patients were contacted by telephone and informed about the study procedures and objectives, and all participants provided informed consent prior to inclusion. The primary objective was to evaluate the global and sexual QoL of women younger than 40 years old with breast cancer who had completed primary treatment for breast cancer at least 12 months prior. We used the EORTC C30 and SHQ-22 questionnaires. The secondary objective was to evaluate the information patients received about fertility preservation. Eligible women were younger than 40 years old, self-declared sexually active, diagnosed with nonmetastatic breast cancer, and had completed surgery as well as chemotherapy and/or radiotherapy when indicated.

### 2.2. Data and Measures

Global health and socio-demographic information, including employment status, physical activity, and partner status, were self-reported by participants. Age at diagnosis, pathology report, type of treatment (type of surgery, radiotherapy, and chemotherapy) and comorbidities were collected from the medical records. Patients were invited to complete the two EORTC questionnaires only once. The general EORTC QLQ-C30 questionnaire dedicated to all cancer patients includes 30 items assessing the global health status with 5 functional scores (physical, role, cognitive, social, and emotional) and 9 symptom scores (nausea and vomiting, pain, fatigue, dyspnea, sleep disturbances, appetite loss, constipation, diarrhea, and financial difficulties). The EORTC SHQ-22 is a multi-dimensional QoL instrument used to measure sexual health in patients with cancer (men or women). This new tool covers both sexual functioning and psychosexual components. It includes 8 items on sexual satisfaction, 3 items on sexual pain, and 11 single items in an integrative approach, leading to 7 functional scales and 4 symptom scales. Following the EORTC scoring manual, items related to sexual functioning and satisfaction were analyzed for all participants, while specific items regarding sexual activity were completed only by those who reported being sexually active in the past four weeks. This distinction was maintained during analysis to ensure that the lack of sexual intercourse—potentially a sign of severe dysfunction—was not misinterpreted as ‘missing data’, but rather integrated into the symptom profile of the cohort. Fertility-related aspects were assessed using a structured 7-item survey developed by a multidisciplinary team of oncologists and reproductive specialists in our institute. The survey was designed to capture specific clinical pathways and counselling practices within the Romanian healthcare system that are not covered by existing validated modules. A pilot test was conducted on a small group of 5 patients to ensure clarity and face validity of the questions prior to study initiation.

### 2.3. Statistical Considerations

Patients were identified from the institutional oncology database, between January 2023 and December 2024. Scores from the two EORTC questionnaires were calculated according to the EORTC Scoring Manuals and are presented as means with standard deviations (SD) and medians with interquartile ranges (IQR), as appropriate.

All the data from the study were analyzed using IBM SPSS Statistics 25 and illustrated using Microsoft Office Excel/Word 2024. Qualitative variables were written as counts or percentages.

Quantitative variables were written as means with standard deviations (along with 95% confidence intervals for means) or medians with interquartile ranges. Normality of the quantitative variables was assessed using the Shapiro–Wilk Test. Quantitative independent variables with non-parametric distribution were tested between groups using the Mann–Whitney U Test/Kruskal–Wallis H Test. Quantitative independent variables with normal distribution were tested between groups using the Student *t*-Test/One-Way ANOVA Test (after verifying the homogeneity of variances using Levene’s test). Effect sizes were also reported for all comparisons in the study (η^2^ for Kruskal–Wallis H/One-Way ANOVA tests, r for Mann–Whitney U tests and Cohen’d for Student *t*-Tests).

The threshold considered for the significance level for all tests was α = 0.05.

Multiple testing corrections were not applied because the parameters were tested strictly against four primary qualitative variables—disease stage, chemotherapy, endocrine therapy, and fertility preservation procedures—and applying such corrections to our limited sample size would significantly increase the risk of Type II errors, potentially masking clinically relevant associations. However, we acknowledge that the varying allocation ratios and lower effect sizes encountered in the post hoc analysis suggest the need for a larger cohort to confirm all observed differences as significant, a constraint that has been explicitly addressed as a study limitation.

## 3. Results

### 3.1. Patient Characteristics

Patients were identified through the institutional electronic database, “Prof. Ion Chiricuta”, Cluj-Napoca, Romania, based on the following inclusion criteria: age < 40 years at diagnosis, stage I–III and histologically confirmed non-metastatic breast cancer, and a minimum of 12 months since the completion of primary treatment (surgery, chemotherapy, or radiotherapy). Of the 134 eligible survivors identified, 65 (48.5%) provided written informed consent and completed the questionnaires. The main reasons for non-participation were lack of response to the invitation by telephone or text message or e-mail (n = 61), explicit decline (n = 5), and deceased status (n = 3). To assess potential selection bias, a comparison between responders and non-responders was performed regarding mean age and disease stage, revealing no statistically significant differences, thus suggesting that the study cohort is representative of the institutional population. Comprehensive socio-demographic and clinical data were collected, including age at diagnosis, time since treatment completion, tumour characteristics (histological subtype, TNM stage), type of systemic therapy (chemotherapy, endocrine therapy, ovarian function suppression), and partnership status at the time of the survey. Data from Table 1 show the characteristics of the analyzed patients. Most of these patients were classified as stage II (50.8%); 92.3% of the patients received chemotherapy, 78.5% radiotherapy and 72.3% endocrine therapy and only 2 patients (3.1%) had a relapse. 60% of the patients were informed regarding fertility issues before treatment; 16.9% of the patients had undergone fertility preservation procedures; and 29.2% of the patients intended to have a pregnancy after treatment.

### 3.2. The EORTC Questionnaires

Data from Table 2 show the description of analyzed scores in the study group. Results show the following:

- For QLQ-C30 scores:
  ◦ In functional scales, the least affected were physical functioning (median = 86.67, IQR = 73.33–93.33) and role functioning (median = 83.33, IQR = 66.67–100), indicating a low level of impairment in these functions. Emotional functioning (Emotional Functioning), cognitive functioning (Cognitive Functioning), and social functioning (Social Functioning) showed score values consistent with a low to moderate level of impairment.
  ◦ In symptom scales, the most frequent symptoms were fatigue (median = 33.33, IQR = 22.22–66.67) and insomnia (median = 33.33, IQR = 33.33–66.67), with financial difficulties also being commonly reported (median = 33.3, IQR = 0–66.7).
  ◦ Median global health score was 66.67 points (IQR = 66.67–83.33) indicating a good status.
- For QLQ-SH22 scores:
  ◦ Regarding the specific aspects assessed by the questionnaire, the most adversely affected domains of sexual life (as indicated by higher score values) were reduced libido (median = 66.7, IQR = 33.3–100), treatment-related impairment of sexual life (median = 66.7, IQR = 33.3–100), inadequate communication with healthcare professionals (median = 100, IQR = 66.7–100), and vaginal dryness (median = 66.7, IQR = 33.3–66.7).
  ◦ Median sexual satisfaction score was 62.5 points (IQR = 40.83–75), indicating a high level of dissatisfaction.
  ◦ Median sexual pain score was 33.33 points (IQR = 0–66.67), indicating a low to moderate level of pain.

**Table 2:** Description of analyzed scores in the study group.

Parameter (QLQ-C30 Scores)	Value (Mean ± SD, 95% C.I. Median (IQR))
Score-PF (Physical functioning)	82.67 ± 15.58, 78.8–86.5, 86.67 (73.33–93.33)
Score-RF (Role functioning)	80 ± 25.03, 73.8–86.2, 83.33 (66.67–100)
Score-EF (Emotional functioning)	61.79 ± 26.71, 55.1–68.4, 66.67 (41.67–83.33)
Score-CF (Cognitive functioning)	65.38 ± 27.05, 58.6–72.1, 66.67 (50–83.33)
Score-SF (Social functioning)	67.69 ± 24.27, 61.6–73.7, 66.67 (66.67–83.33)
Score-Fatigue	44.62 ± 27.32, 37.8–51.4, 33.33 (22.22–66.67)
Score-Nausea	13.08 ± 17.55, 8.7–17.4, 0 (0–16.67)
Score-Pain	34.87 ± 26.47, 28.3–41.4, 33.33 (16.67–50)
Score-Dyspnea	17.95 ± 26.4, 11.4–24.5, 0 (0–33.33)
Score-Insomnia	43.59 ± 31.69, 35.7–51.4, 33.33 (33.33–66.67)
Score-Appetite loss	16.41 ± 28.33, 9.4–23.4, 0 (0–33.33)
Score-Constipation	22.56 ± 27.7, 15.7–29.4, 0 (0–33.33)
Score-Diarrhea	19.49 ± 29.4, 12.2–26.7, 0 (0–33.33)
Score-Financial difficulties	31.28 ± 31.66, 23.4–39.1, 33.33 (0–66.67)
Score-GH (Global health)	68.33 ± 17.25, 64–72.6, 66.67 (66.67–83.33)
QLQ-SH22 scores	
Score-SA (Sexual activity)	50.26 ± 25.76, 43.8–56.6, 33.33 (33.33–66.67)
Score-DL (Decreased libido)	64.1 ± 34, 55.6–72.5, 66.67 (33.33–100)
Score-IN (Incontinence)	29.23 ± 27.32, 22.4–36, 33.33 (0–50)
Score-FA (Fatigue)	50.77 ± 34.41, 42.2–59.3, 33.33 (33.33–66.67)
Score-TR (Treatment)	58.46 ± 34.87, 49.8–67.1, 66.67 (33.33–100)
Score-CO (Communication with professionals)	81.54 ± 23.59, 75.7–87.3, 100 (66.67–100)
Score-PA (Partnership)	43.08 ± 34.72, 34.4–51.6, 33.33 (0–66.67)
Score-BI (Body image)	50.26 ± 34.42, 41.7–58.8, 33.33 (33.33–66.67)
Score-VD (Vaginal dryness)	51.02 ± 23.67, 44.2–57.8, 66.67 (33.33–66.67)
Score-SS (Sexual satisfaction)	60.95 ± 22.56, 55.3–66.5, 62.5 (40.83–75)
Score-SP (Sexual pain)	38.72 ± 33.56, 30.4–47, 33.33 (0–66.67)

Stratified analyses according to disease stage and chemotherapy exposure revealed no statistically significant differences in any of the analyzed scores (all *p* > 0.05), as shown in Table 3 and Table 4.

**Figure 1:**
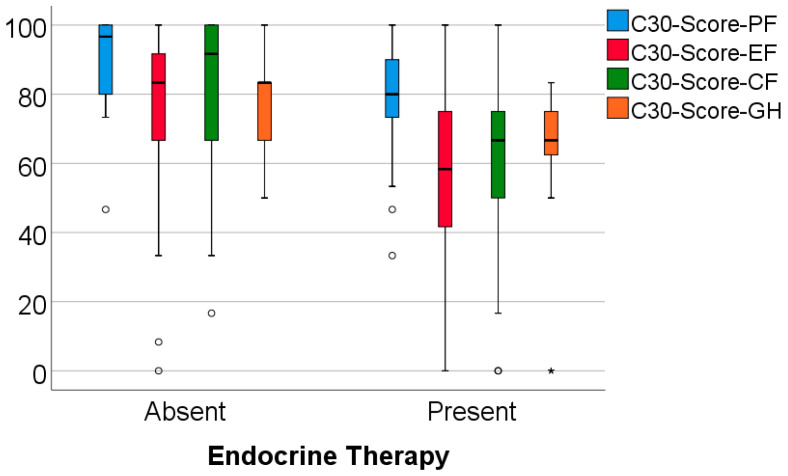
Comparison of QLQ-C30 physical functioning (PF), emotional functioning (EF), cognitive functioning (CF) and global health (GH) scores according to the existence of endocrine therapy.

**Figure 2:**
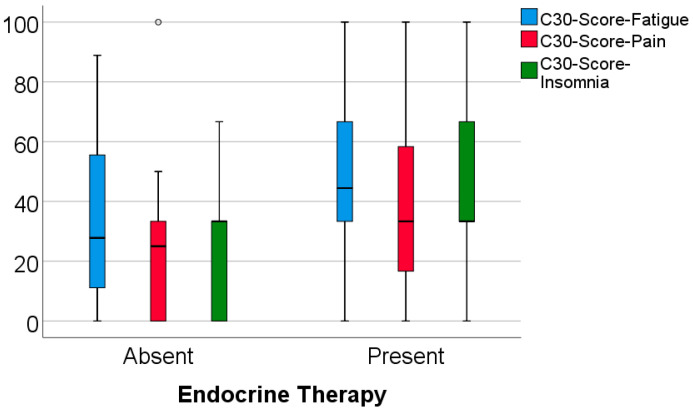
Comparison of QLQ-C30 fatigue, pain and insomnia scores according to the existence of endocrine therapy.

**Figure 3:**
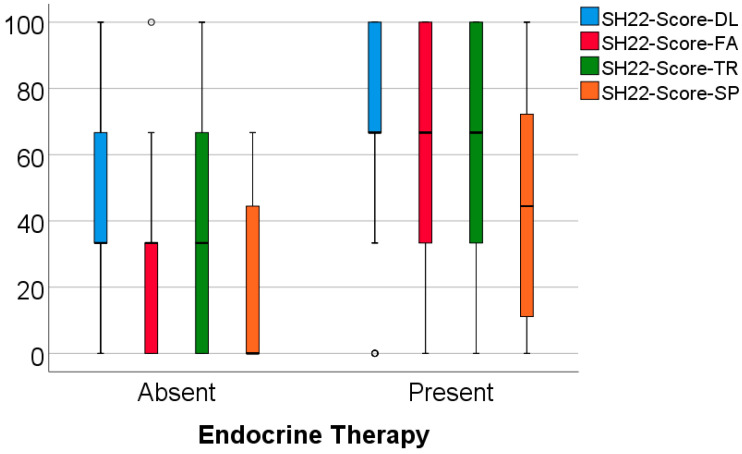
Comparison of QLQ-SH22 decreased libido (DL), fatigue (FA), treatment (TR) and sexual pain (SP) scores according to the existence of endocrine therapy.

Data from Table 5 and Figure 1, Figure 2 and Figure 3 show the comparison of analyzed scores according to the existence of endocrine therapy. Significant differences between groups were observed for the following scores:

- In case of QLQ-C30 functional scales, patients with endocrine therapy had significantly more issues (expressed by lower functional scores) regarding physical functioning (median = 80, IQR = 73.3–93.3 vs. median = 96.7, IQR = 80–100, *p* = 0.006), emotional functioning (median = 58.3, IQR = 41.67–75 vs. median = 83.3, IQR = 62.5–93.75, *p* = 0.025) and cognitive functioning (median = 66.7, IQR = 50–83.33 vs. median = 91.67, IQR = 66.7–100, *p* = 0.001) than patients without endocrine therapy;
- In case of QLQ-C30 symptom scales, patients with endocrine therapy had significantly more issues (expressed by higher symptom scores) regarding fatigue (median = 44.4, IQR = 33.3–66.7 vs. median = 27.78, IQR = 11.1–55.5, *p* = 0.009), pain (median =33.3, IQR = 16.7–66.7 vs. median = 25, IQR = 0–37.5, *p* = 0.035) and insomnia (median = 33.3, IQR = 33.3–66.7 vs. median = 33.3, IQR = 0–33.3, *p* = 0.002) than patients without endocrine therapy;
- Overall, patients with endocrine therapy reported lower global health (median = 66.7, IQR = 58.3–75 vs. median = 83.3, IQR = 66.7–83.3, *p* = 0.020);
- In case of QLQ-SH22 sexual health scores, patients with endocrine therapy had significantly more issues (expressed by higher scores) regarding decreased libido (DL) (median = 66.7, IQR = 66.7–100 vs. median = 33.3, IQR = 25–66.7, *p* = 0.009), fatigue (FA) (median = 66.7, IQR = 33.3–100 vs. median = 33.3, IQR = 0–41.67, *p* = 0.005), treatment (TR) (median = 66.7, IQR = 33.3–100 vs. median = 33.3, IQR = 0–66.7, *p* = 0.005) and sexual pain (median = 44.4, IQR = 11.1–77.7 vs. median = 0, IQR = 0–44.4, *p* = 0.002).

**Table 5:** Comparison of analyzed scores according to the existence of endocrine therapy.

Endocrine Therapy/Score		Absent	Present	*p* *
Score-PF	Mean ± SD	90 ± 14.32	79.86 ± 15.26	0.006 r = 0.341
	95% C.I.	82.88–97.12	75.38–84.34	
	Median (IQR)	96.7 (80–100)	80 (73.3–93.3)	
Score-RF	Mean ± SD	84.26 ± 28.28	78.37 ± 23.8	0.138 r = 0.184
	95% C.I.	70.2–98.32	71.38–85.36	
	Median (IQR)	100 (66.7–100)	83.3 (66.7–100)	
Score-EF	Mean ± SD	71.3 ± 30.41	58.16 ± 24.54	0.025 r = 0.277
	95% C.I.	56.17–86.42	50.95–65.36	
	Median (IQR)	83.3 (62.5–93.75)	58.3 (41.67–75)	
Score-CF	Mean ± SD	81.48 ± 24.84	59.22 ± 25.49	0.001 r = 0.401
	95% C.I.	69.13–93.84	51.74–66.7	
	Median (IQR)	91.67 (66.7–100)	66.7 (50–83.33)	
Score-SF	Mean ± SD	73.15 ± 27.5	65.6 ± 22.89	0.088 r = 0.211
	95% C.I.	59.47–86.82	58.88–72.33	
	Median (IQR)	75 (66.7–100)	66.7 (66.7–66.7)	
Score-Fatigue	Mean ± SD	30.86 ± 23.97	49.88 ± 26.9	0.009 r = 0.325
	95% C.I.	18.94–42.78	41.98–57.78	
	Median (IQR)	27.78 (11.1–55.5)	44.4 (33.3–66.7)	
Score-Nausea	Mean ± SD	12.04 ± 17.9	13.48 ± 17.59	0.771 r = 0.036
	95% C.I.	3.14–20.94	8.31–18.64	
	Median (IQR)	0 (0–16.7)	0 (0–16.7)	
Score-Pain	Mean ± SD	24.07 ± 27.54	39.01 ± 25.12	0.035 r = 0.262
	95% C.I.	10.37–37.77	31.63–46.38	
	Median (IQR)	25 (0–37.5)	33.3 (16.7–66.7)	
Score-Dyspnea	Mean ± SD	11.1 ± 22.86	20.57 ± 27.41	0.136 r = 0.185
	95% C.I.	0–22.48	12.52–28.62	
	Median (IQR)	0 (0–8.33)	0 (0–33.3)	
Score-Insomnia	Mean ± SD	24.07 ± 25.06	51.06 ± 30.96	0.002 r = 0.38
	95% C.I.	11.61–36.54	41.97–60.16	
	Median (IQR)	33.3 (0–33.3)	33.3 (33.3–66.7)	
Score-Appetite	Mean ± SD	16.7 ± 28.58	16.31 ± 28.55	0.922 r = 0.012
	95% C.I.	2.45–30.88	7.93–24.69	
	Median (IQR)	0 (0–33.3)	0 (0–33.3)	
Score-Constipation	Mean ± SD	16.7 ± 28.58	24.82 ± 27.33	0.162 r = 0.173
	95% C.I.	2.45–30.88	16.8–32.85	
	Median (IQR)	0 (0–33.3)	33.3 (0–33.3)	
Score-Diarrhea	Mean ± SD	14.81 ± 23.49	21.28 ± 31.41	0.543 r = 0.075
	95% C.I.	3.13–26.5	12.05–30.5	
	Median (IQR)	0 (0–33.3)	0 (0–33.3)	
Score-FD	Mean ± SD	25.93 ± 31.42	33.3 ± 31.85	0.364 r = 0.112
	95% C.I.	10.3–41.55	23.98–42.7	
	Median (IQR)	16.7 (0–41.67)	33.3 (0–66.7)	
Score-GH	Mean ± SD	75.46 ± 14.42	65.6 ± 17.6	0.020 r = 0.288
	95% C.I.	68.3–82.64	60.43–70.77	
	Median (IQR)	83.3 (66.7–83.3)	66.7 (58.3–75)	
Score-SA	Mean ± SD	57.41 ± 22.3	47.52 ± 26.69	0.173 r = 0.169
	95% C.I.	46.32–68.50	39.68–55.35	
	Median (IQR)	66.7 (33.3–66.7)	33.3 (33.3–66.7)	
Score-DL	Mean ± SD	46.3 ± 34.56	70.92 ± 31.56	0.009 r = 0.323
	95% C.I.	29.11–63.48	61.66–80.19	
	Median (IQR)	33.3 (25–66.7)	66.7 (66.7–100)	
Score-IN	Mean ± SD	20.37 ± 23.26	32.62 ± 28.22	0.116 r = 0.194
	95% C.I.	8.8–31.94	24.34–40.91	
	Median (IQR)	16.7 (0–33.3)	33.3 (0–66.7)	
Score-FA	Mean ± SD	31.48 ± 29.08	58.16 ± 33.67	0.005 r = 0.349
	95% C.I.	17.02–45.95	48.27–68.04	
	Median (IQR)	33.3 (0–41.67)	66.7 (33.3–100)	
Score-TR	Mean ± SD	38.9 ± 32.84	65.96 ± 32.96	0.005 r = 0.347
	95% C.I.	22.56–55.22	56.28–75.64	
	Median (IQR)	33.3 (0–66.7)	66.7 (33.3–100)	
Score-CO	Mean ± SD	83.3 ± 20.61	80.85 ± 24.81	0.856 r = 0.022
	95% C.I.	73.08–93.58	73.57–88.14	
	Median (IQR)	100 (66.7–100)	100 (66.7–100)	
Score-PA	Mean ± SD	29.63 ± 32.11	48.23 ± 34.62	0.053 r = 0.239
	95% C.I.	13.66–45.6	38.06–58.4	
	Median (IQR)	33.3 (0–66.7)	33.3 (33.3–66.7)	
Score-BI	Mean ± SD	44.4 ± 32.33	52.48 ± 35.26	0.373 r = 0.110
	95% C.I.	28.36–60.53	42.13–62.84	
	Median (IQR)	33.3 (33.3–66.7)	66.7 (33.3–66.7)	
Score-VD	Mean ± SD	47.62 ± 21.54	52.38 ± 24.63	0.491 r = 0.098
	95% C.I.	35.18–60.06	43.92–60.84	
	Median (IQR)	50 (33.3–66.7)	66.7 (33.3–66.7)	
Score-SS	Mean ± SD	56.11 ± 21.33	62.8 ± 22.97	0.132 r = 0.187
	95% C.I.	45.5–66.72	56.06–69.55	
	Median (IQR)	50.4 (36.4–67.7)	66.7 (45.8–79.1)	
Score-SP	Mean ± SD	18.52 ± 24.7	46.45 ± 33.48	0.002 r = 0.388
	95% C.I.	6.24–30.8	36.62–56.28	
	Median (IQR)	0 (0–44.4)	44.4 (11.1–77.7)	

* Mann–Whitney U Test.

**Figure 4:**
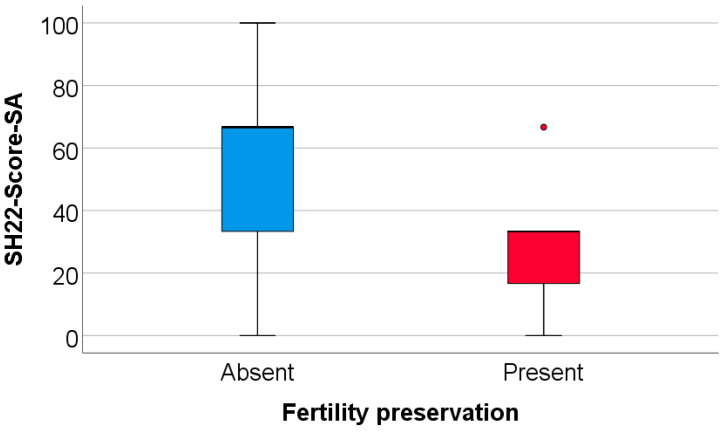
Comparison of QLQ-SH22 sexual activity (SA) score according to the existence of fertility preservation procedure.

Data from Table 6 and Figure 4 show the comparison of analyzed scores according to the existence of fertility preservation procedure. Significant differences between groups were observed only for the sexual activity score (*p* = 0.015) of patients that had undergone fertility preservation procedures and had significantly fewer issues regarding sexual activity (expressed by lower scores) (median = 33.3, IQR = 0–33.3 vs. median = 66.7, IQR = 33.3–100) in comparison to patients without these procedures.

### 3.3. The Fertility Preservation Questionnaire

Thirty-nine of 65 patients (60%) reported receiving information from their medical oncologist regarding fertility preservation. The options discussed included oocyte cryopreservation (24.6%), embryo cryopreservation (1%), or both options (15.4%). Conversely, 26 patients (40%) stated that they had received no information or insufficient information to enable informed decision-making. In some cases, fertility preservation may be briefly mentioned during the oncology consultation without detailed counselling or referral to a fertility specialist, thereby limiting patients’ understanding of individualized fertility preservation strategies. Eleven patients underwent in vitro fertilization procedures, while 19 patients expressed an intention to pursue a future pregnancy. In contrast, 46 patients reported no intention to conceive, most frequently citing the reason: “I already have one or two children.” For some patients, prioritization of oncologic treatment was the primary determinant, while religious considerations were also reported as influencing factors.

## 4. Discussion

This study provides evidence for global and sexual quality of life among young women diagnosed with breast cancer, while also addressing the current availability and quality of oncofertility counselling in Romania. By focusing on patients younger than 40 years old who had completed primary cancer treatment at least one year prior, our analysis captures long-term survivorship-related challenges that extend beyond the acute treatment phase. Despite its unicentric design, the study was performed in a national oncology referral centre, which strengthens the clinical significance of the results and may support their extrapolation to similar tertiary care environments.

In our cohort of young breast cancer survivors, sexual health emerged as a substantially impaired domain despite relatively preserved global health status. Although median global health scores suggested an overall satisfactory quality of life, several functional domains—particularly physical, role, emotional, and cognitive functioning—remained compromised. Fatigue and insomnia were the most prominent symptoms reported. These findings are consistent with previous evidence indicating that younger survivors often experience greater psychosocial and functional challenges compared with older women [27], potentially related to life-stage factors such as professional activity, childcare responsibilities, premenopausal status at diagnosis, and concerns regarding fertility and treatment-induced menopause [28].

Sexual dysfunction was common in our cohort, with reduced libido, vaginal dryness, and treatment-related impairment of sexual life representing the most severely affected domains. Breast cancer itself confers an increased risk of sexual dysfunction, including dyspareunia, vaginal dryness, vaginal complications, and reduced libido. This excess risk was most pronounced within the first 5 years following diagnosis and among women younger than 50 years at the time of diagnosis. Exposure to radiotherapy, chemotherapy, and endocrine therapy was associated with a higher likelihood of sexual function symptoms, whereas a higher baseline body mass index appeared to confer a lower risk [8].

Endocrine therapy was one of the variables most clearly associated with lower quality of life scores. Patients reported worse physical, emotional, and cognitive functioning, as well as increased symptom burden, including fatigue, pain, and insomnia. However, given the cross-sectional nature of this study, a direct causal relationship cannot be definitively established. These differences may also be influenced by underlying factors such as tumour stage or the cumulative effects of previous chemotherapy. In addition, endocrine therapy was associated with more pronounced sexual dysfunction, particularly decreased libido, treatment-related sexual impairment, and sexual pain. These findings are in line with the existing literature describing the negative impact of endocrine treatment on menopausal symptoms, sexual functioning, and quality of life, and highlight the need for proactive symptom management and supportive interventions in this subgroup. Chang et al. demonstrated that endocrine therapy was associated with a 1.46-fold higher likelihood of a sexual dysfunction diagnosis, whereas radiotherapy was associated with a 1.17-fold increased risk [8].

Communication about sexual health with healthcare professionals (median score 100) was difficult in this cohort and represents a critical failure in survivorship care. The critical gap aligns with broader evidence indicating that while sexual dysfunction affects the majority of young breast cancer survivors, up to 73% of patients report a total lack of information from their healthcare team [29]. International guidelines, including those from ASCO and NCCN, emphasize that clinicians must proactively initiate discussions regarding sexual well-being, utilizing structured frameworks like the PLISSIT model to normalize these concerns [30]. This may help normalize these conversations and reduce stigma, and it is particularly vital for young survivors, who face a unique constellation of challenges—including treatment-induced amenorrhea, body image distress, and the intensification of symptoms by endocrine therapy—which directly correlate with inferior quality of life. Standardized instruments recommended for clinical use include the Female Sexual Function Index (FSFI), the Brief Sexual Symptom Checklist (adapted for female cancer patients), the Arizona Sexual Experience Scale (ASEX), and the PROMIS-1 item screener [31]. Despite the availability of validated screening tools and evidence-based interventions ranging from non-hormonal lubricants to cognitive-behavioural therapy, systemic barriers such as clinician discomfort and time constraints continue to foster a ‘clinical silence’ [31]. Consequently, survivors are increasingly forced to seek information from non-clinical sources, such as social media, highlighting an urgent need to integrate standardized sexual health assessments into routine oncological follow-up.

In certain socio-cultural contexts, open discussions about sexuality are not actively encouraged, further reinforcing this withdrawal. In Romania, sexual satisfaction remains a deeply rooted taboo, a silence that often persists even within the clinical setting. This cultural barrier is further exacerbated by systemic pressures in public hospitals, where the overwhelming volume of patients leaves clinicians with extremely limited time, which is almost exclusively dedicated to urgent oncological treatment strategies. In such circumstances, healthcare professionals bear an even greater responsibility to systematically assess sexual health and to initiate and sustain discussions when clinically appropriate, ensuring that quality-of-life concerns are not entirely eclipsed by the immediate demands of cancer care [32,33].

Importantly, the present study also points to persistent gaps in oncofertility counselling. Although 60% of patients reported receiving some information regarding fertility preservation prior to treatment, a substantial proportion (40%) indicated that this medical option appeared to be mentioned briefly during oncology consultations, without detailed discussion or referral to reproductive medicine specialists. Only a minority of patients underwent fertility preservation procedures, while nearly one-third expressed an intention to pursue a future pregnancy. Conversely, the majority reported no desire for future childbearing, most commonly because they had already completed their families.

The counselling rate observed in our cohort is comparable to the incomplete implementation reported in other healthcare systems. Large-scale data from the United States suggest that approximately 44% of reproductive-aged cancer patients receive fertility-related counselling prior to treatment, with higher rates observed among women than men [34]. European population-based studies report similar or lower rates, with counselling frequencies ranging between 30% and 50% [35]. Although targeted quality improvement initiatives have demonstrated that counselling rates can exceed 80% when standardized referral pathways and educational interventions are implemented, such approaches are not yet routinely integrated into everyday oncology practice [36].

In the Romanian healthcare context, these may include a lack of specialized oncofertility units, the high out-of-pocket costs for oocyte cryopreservation which are not reimbursed, and cultural taboos surrounding reproductive technology. Furthermore, the ‘treatment priority’ mindset—where the urgency of oncology care overshadows future fertility—remains a dominant factor. Integrating a multidisciplinary ‘oncofertility navigator’ into the standard care pathway could mitigate these organizational and psychological barriers.

The strengths of this study include its focus on a young, understudied patient population, the use of validated EORTC instruments to assess both global and sexual quality of life, and the integration of patient-reported data on fertility preservation counselling. However, several constraints must be acknowledged. The single-centre design and relatively small sample size may limit generalizability. Furthermore, the cross-sectional nature of the study precludes causal inferences, and self-reported questionnaires may be subject to recall and response bias. Regarding statistical constraints, although an a priori power analysis indicated a minimum sample size of 60, the exploratory nature of this study and the varying allocation ratios in subgroup analyses meant that multiple testing corrections were not applied to avoid an increased risk of Type II errors. Consequently, some observed differences may require confirmation in larger cohorts. Moreover, while the fertility preservation survey was pilot-tested for face validity, it lacks formal psychometric validation. Future research employing qualitative methods, such as focus group interviews, is recommended to further explore these barriers. Ultimately, larger prospective studies are required to confirm these preliminary observations and to develop targeted, evidence-based interventions that address the systemic communication barriers identified in this evaluation.

## 5. Conclusions

Young breast cancer survivors maintain a satisfactory functional status but face significant long-term challenges regarding sexual health and symptomatic burden. Our findings highlight an alarming communication gap; sexual health and fertility remain largely unaddressed in routine oncologic follow-up. Existing literature often overlooks the specific needs of Eastern European populations, such as those in Romania, where healthcare infrastructure and cultural taboos may differ from Western models. There is a notable gap in understanding how systemic barriers and a lack of specialized oncofertility units impact the quality of life for young survivors. In conclusion, there is an urgent need to dismantle the barriers of clinical silence by integrating mandatory sexual health and fertility assessments into standard survivorship protocols, ensuring that ‘cure’ does not come at the expense of ‘quality of life’.

## Figures and Tables

**Table 1:** Characteristics of the analyzed patients.

Parameter	Value (Nr., %)
Disease stage	
Stage I	10 (15.4%)
Stage II	33 (50.8%)
Stage III	22 (33.8%)
Chemotherapy	60 (92.3%)
Radiotherapy	51 (78.5%)
Endocrine therapy	47 (72.3%)
Relapse	2 (3.1%)
Fertility informing before treatment	39 (60%)
Fertility preservation procedure	11 (16.9%)
Pregnancy intention	19 (29.2%)

**Table 3:** Comparison of analyzed scores according to the stage disease.

Stage/Score		Stage I	Stage II	Stage III	*p* *
Score-PF	Mean ± SD	86 ± 18.17	83.03 ± 11.67	80.61 ± 19.5	0.539 η^2^ = 0
	95% C.I.	73–99	78.9–87.1	71.9–89.2	
	Median (IQR)	93.3 (76.6–100)	86.6 (73.3–93.3)	86.6 (70–95)	
Score-RF	Mean ± SD	81.67 ± 16.57	82.83 ± 21.03	75 ± 32.83	0.861 η^2^ = 0
	95% C.I.	69.8–93.5	75.3–90.3	60.4–89.5	
	Median (IQR)	75 (66.7–100)	83.3 (66.7–100)	91.6 (62.5–100)	
Score-EF	Mean ± SD	50 ± 34.47	65.4 ± 22.06	61.74 ± 28.94	0.392 η^2^ = 0
	95% C.I.	25.3–74.6	57.5–73.2	58.9–74.5	
	Median (IQR)	45.8 (27–75)	66.7 (45.8–83.3)	75 (47.9–83.3)	
Score-CF	Mean ± SD	70 ± 33.14	63.13 ± 24.91	66.7 ± 28.17	0.550 η^2^ = 0
	95% C.I.	46.3–93.7	54.3–71.9	54.1–79.1	
	Median (IQR)	83.3 (45.8–100)	66.7 (50–83.3)	66.7 (50–87.5)	
Score-SF	Mean ± SD	66.7 ± 28.32	69.2 ± 20.46	65.91 ± 28.4	0.972 η^2^ = 0
	95% C.I.	46.4–86.9	61.9–76.4	53.3–78.5	
	Median (IQR)	66.7 (45.8–100)	66.7 (66.7–83.3)	66.7 (62.5–83.3)	
Score-Fatigue	Mean ± SD	34.44 ± 32.05	47.47 ± 21.74	44.95 ± 32.43	0.366 η^2^ = 0
	95% C.I.	11.5–57.3	39.7–55.1	30.5–59.3	
	Median (IQR)	27.7 (8.33–55.5)	44.4 (33.3–66.7)	38.9 (22.2–77.7)	
Score-Nausea	Mean ± SD	8.33 ± 8.78	14.14 ± 17.24	13.64 ± 20.98	0.819 η^2^ = 0
	95% C.I.	2.05–14.62	8.03–20.25	4.33–22.94	
	Median (IQR)	8.33 (0–16.7)	0 (0–33.3)	0 (0–16.7)	
Score-Pain	Mean ± SD	26.67 ± 23.83	37.37 ± 19.1	34.85 ± 35.97	0.324 η^2^ = 0.004
	95% C.I.	9.62–43.71	30.6–44.15	18.9–50.8	
	Median (IQR)	25 (0–50)	33.3 (33.3–50)	25 (0–66.7)	
Score-Dyspnea	Mean ± SD	23.3 ± 35.31	14.1 ± 18.7	21.2 ± 31.78	0.888 η^2^ = 0
	95% C.I.	0–48.6	7.51–20.77	7.12–35.3	
	Median (IQR)	0 (0–41.67)	0 (0–33.3)	0 (0–41.67)	
Score-Insomnia	Mean ± SD	43.3 ± 38.65	48.48 ± 28.97	36.36 ± 32.38	0.350 η^2^ = 0.001
	95% C.I.	15.6–70.9	38.2–58.7	22–50.7	
	Median (IQR)	33.3 (0–75)	33.3 (33.3–66.7)	33.3 (0–66.7)	
Score-Appetite	Mean ± SD	16.67 ± 36	15.15 ± 25.12	18.18 ± 30.39	0.816 η^2^ = 0
	95% C.I.	0–42.4	6.24–24.06	4.71–31.66	
	Median (IQR)	0 (0–16.7)	0 (0–33.3)	0 (0–33.3)	
Score-Constipation	Mean ± SD	20 ± 28.11	24.24 ± 26.7	21.21 ± 30.07	0.745 η^2^ = 0
	95% C.I.	0–40.11	14.77–33.71	7.88–34.54	
	Median (IQR)	0 (0–41.67)	33.3 (0–33.3)	0 (0–33.3)	
Score-Diarrhea	Mean ± SD	16.67 ± 23.57	16.16 ± 27.79	25.76 ± 34.01	0.545 η^2^ = 0
	95% C.I.	0–33.53	6.31–26.02	10.68–40.84	
	Median (IQR)	0 (0–33.33)	0 (0–33.33)	0 (0–41.67)	
Score-FD	Mean ± SD	20 ± 28.1	30.31 ± 28.1	37.88 ± 37.51	0.398 η^2^ = 0
	95% C.I.	0–40.11	20.34–40.26	21.25–54.51	
	Median (IQR)	0 (0–41.67)	33.3 (0–50)	33.3 (0–66.7)	
Score-GH	Mean ± SD	65.83 ± 28.17	69.19 ± 11.31	68.18 ± 19.18	0.909 η^2^ = 0
	95% C.I.	45.68–86	65.18–73.2	59.68–76.7	
	Median (IQR)	70.83 (50–85.4)	66.7 (66.7–83.3)	70.8 (64.5–83.3)	
Score-SA	Mean ± SD	56.67 ± 22.49	48.48 ± 27.75	50 ± 24.66	0.697 η^2^ = 0
	95% C.I.	40.57–72.76	38.64–58.33	39.06–60.94	
	Median (IQR)	66.7 (33.3–66.7)	33.3 (33.3–66.7)	33.3 (33.3–66.7)	
Score-DL	Mean ± SD	56.67 ± 41.72	69.7 ± 30.46	59.09 ± 35.53	0.488 η^2^ = 0
	95% C.I.	26.82–86.51	58.9–80.5	43.34–74.85	
	Median (IQR)	66.7 (0–100)	66.7 (50–100)	66.7 (33.3–100)	
Score-IN	Mean ± SD	23.33 ± 22.5	35.35 ± 30	22.7 ± 23.87	0.238 η^2^ = 0.014
	95% C.I.	7.24–39.43	24.72–45.98	12.14–33.31	
	Median (IQR)	33.3 (0–33.33)	33.33 (0–66.7)	33.3 (0–33.3)	
Score-FA	Mean ± SD	56.7 ± 41.72	53.54 ± 34.3	43.9 ± 31.51	0.475 η^2^ = 0
	95% C.I.	26.82–86.51	41.37–65.7	29.97–57.91	
	Median (IQR)	66.7 (0–100)	66.7 (33.3–83.3)	33.3 (33.3–66.7)	
Score-TR	Mean ± SD	53.3 ± 42.16	59.6 ± 34.11	59.09 ± 34.01	0.938 η^2^ = 0
	95% C.I.	23.17–83.5	47.5–71.7	44.01–74.17	
	Median (IQR)	66.7 (0–100)	66.7 (33.3–100)	66.7 (33.3–100)	
Score-CO	Mean ± SD	73.3 ± 34.42	83.84 ± 20.61	81.82 ± 22.36	0.758 η^2^ = 0
	95% C.I.	48.71–97.96	76.53–91.15	71.9–91.73	
	Median (IQR)	83.3 (58.3–100)	100 (66.7–100)	100 (66.7–100)	
Score-PA	Mean ± SD	40 ± 34.42	47.4 ± 36.35	37.8 ± 33	0.614 η^2^ = 0
	95% C.I.	15.37–64.63	34.58–60.37	23.24–52.51	
	Median (IQR)	33.3 (0–66.7)	33.3 (16.7–66.7)	33.3 (0–66.7)	
Score-BI	Mean ± SD	56.7 ± 41.72	50.5 ± 34.48	46.97 ± 31.97	0.720 η^2^ = 0
	95% C.I.	26.82–86.51	38.28–62.73	32.79–61.14	
	Median (IQR)	66.7 (0–100)	33.3 (33.3–66.7)	33.3 (33.3–66.7)	
Score-VD	Mean ± SD	46.7 ± 18.25	51.28 ± 23.53	51.85 ± 26.12	0.813 η^2^ = 0
	95% C.I.	24–69.34	41.78–60.8	38.86–64.84	
	Median (IQR)	33.3 (33.3–66.7)	66.7 (33.3–66.7)	66.7 (33.3–66.7)	
Score-SS	Mean ± SD	70.33 ± 22.92	60.61 ± 23.27	57.2 ± 21.08	0.314 ** η^2^ = 0.037
	95% C.I.	53.94–86.73	52.36–68.86	47.85–66.55	
	Median (IQR)	74.58 (50–88.3)	66.7 (37.5–75)	62.5 (39.3–70.8)	
Score-SP	Mean ± SD	47.22 ± 39.65	42.42 ± 31.72	29.29 ± 32.8	0.294 η^2^ = 0.007
	95% C.I.	18.86–75.59	31.17–53.67	14.75–43.84	
	Median (IQR)	44.4 (0–87.5)	44.4 (11.1–66.7)	11.1 (0–50)	

* Kruskal–Wallis H Test, ** One-Way ANOVA Test.

**Table 4:** Comparison of analyzed scores according to the existence of chemotherapy.

Chemotherapy/Score		Absent	Present	*p* *
Score-PF	Mean ± SD	82.67 ± 21.91	82.67 ± 15.19	0.729 r = 0.045
	95% C.I.	55.46–100	78.74–86.6	
	Median (IQR)	86.67 (63.33–100)	86.67 (73.33–93.33)	
Score-RF	Mean ± SD	80 ± 18.25	80 ± 25.63	0.765 r = 0.040
	95% C.I.	57.33–100	73.38–86.62	
	Median (IQR)	66.7 (66.7–100)	83.33 (66.7–100)	
Score-EF	Mean ± SD	45 ± 41.5	63.2 ± 25.13	0.305 r = 0.130
	95% C.I.	0–96.53	56.7–69.7	
	Median (IQR)	50 (4.17–83.33)	66.7 (41.67–83.33)	
Score-CF	Mean ± SD	66.7 ± 40.82	65.28 ± 26.1	0.658 r = 0.057
	95% C.I.	15.9–100	58.5–72	
	Median (IQR)	66.7 (33.3–100)	66.7 (50–83.33)	
Score-SF	Mean ± SD	73.33 ± 27.89	67.22 ± 24.15	0.641 r = 0.063
	95% C.I.	38.7–100	61–73.46	
	Median (IQR)	66.7 (50–100)	66.7 (66.7–83.33)	
Score-Fatigue	Mean ± SD	46.67 ± 38	44.44 ± 26.67	0.952 r = 0.009
	95% C.I.	0–93.86	37.55–51.33	
	Median (IQR)	55.56 (11.1–77.7)	33.3 (25–66.7)	
Score-Nausea	Mean ± SD	10 ± 9.13	13.33 ± 18.1	0.952 r = 0.01
	95% C.I.	0–21.33	8.66–18.01	
	Median (IQR)	16.67 (0–16.67)	0 (0–16.67)	
Score-Pain	Mean ± SD	33.3 ± 26.35	35 ± 26.7	1.000 r = 0.001
	95% C.I.	0.61–66.05	28.1–41.9	
	Median (IQR)	33.3 (8.33–58.33)	33.3 (16.7–50)	
Score-Dyspnea	Mean ± SD	40 ± 43.46	16.11 ± 24.15	0.231 r = 0.176
	95% C.I.	0–93.96	9.87–22.35	
	Median (IQR)	33.3 (0–83.33)	0 (0–33.33)	
Score-Insomnia	Mean ± SD	60 ± 43.46	42.22 ± 30.6	0.329 r = 0.131
	95% C.I.	6.04–100	34.32–50.13	
	Median (IQR)	66.7 (16.67–100)	33.3 (33.3–66.7)	
Score-Appetite	Mean ± SD	40 ± 43.46	14.44 ± 26.3	0.186 r = 0.203
	95% C.I.	0–93.96	7.65–21.24	
	Median (IQR)	33.3 (0–83.33)	0 (0–33.3)	
Score-Constipation	Mean ± SD	46.67 ± 44.72	20.56 ± 25.37	0.212 r = 0.174
	95% C.I.	0–100	14–27.11	
	Median (IQR)	66.7 (0–83.33)	0 (0–33.33)	
Score-Diarrhea	Mean ± SD	6.67 ± 14.9	20.56 ± 30.12	0.407 r = 0.121
	95% C.I.	0–25.18	12.77–28.34	
	Median (IQR)	0 (0–16.67)	0 (0–33.33)	
Score-FD	Mean ± SD	26.67 ± 27.89	31.67 ± 32.14	0.839 r = 0.027
	95% C.I.	0–61.3	23.36–39.97	
	Median (IQR)	33.3 (0–50)	33.3 (0–66.7)	
Score-GH	Mean ± SD	75 ± 18.63	67.78 ± 17.18	0.494 r = 0.091
	95% C.I.	51.86–98.14	63.34–72.22	
	Median (IQR)	75 (58.3–91.67)	66.7 (66.7–83.3)	
Score-SA	Mean ± SD	53.33 ± 18.25	50 ± 26.4	0.747 r = 0.044
	95% C.I.	30.66–76	43.18–56.82	
	Median (IQR)	66.7 (33.3–66.7)	33.3 (33.3–66.7)	
Score-DL	Mean ± SD	66.7 ± 40.82	63.9 ± 33.77	0.802 r = 0.033
	95% C.I.	15.98–100	55.16–72.61	
	Median (IQR)	66.7 (33.3–100)	66.7 (33.3–100)	
Score-IN	Mean ± SD	26.67 ± 27.89	29.44 ± 27.51	0.877 r = 0.022
	95% C.I.	0–61.3	22.34–36.55	
	Median (IQR)	33.3 (0–50)	33.3 (0–58.33)	
Score-FA	Mean ± SD	60 ± 36.51	50 ± 34.44	0.510 r = 0.087
	95% C.I.	14.66–100	41.1–58.9	
	Median (IQR)	66.7 (33.3–83.33)	33.3 (33.3–66.7)	
Score-TR	Mean ± SD	60 ± 43.46	58.33 ± 34.51	0.877 r = 0.022
	95% C.I.	6.04–100	49.42–67.25	
	Median (IQR)	66.7 (16.67–100)	66.7 (33.3–100)	
Score-CO	Mean ± SD	80 ± 44.72	81.67 ± 21.63	0.541 r = 0.089
	95% C.I.	24.47–100	76.08–87.26	
	Median (IQR)	100 (50–100)	100 (66.7–100)	
Score-PA	Mean ± SD	40 ± 43.46	43.33 ± 34.33	0.821 r = 0.031
	95% C.I.	0–93.96	34.46–52.20	
	Median (IQR)	33.3 (0–83.33)	33.3 (0–66.7)	
Score-BI	Mean ± SD	40 ± 43.46	51.11 ± 33.87	0.525 r = 0.085
	95% C.I.	0–93.96	42.36–59.86	
	Median (IQR)	33.3 (0–83.33)	33.3 (33.3–66.7)	
Score-VD	Mean ± SD	44.4 ± 19.24	51.45 ± 24.04	0.565 r = 0.096
	95% C.I.	0–92.25	44.31–58.6	
	Median (IQR)	33.3 (33.3–50)	66.7 (33.3–66.7)	
Score-SS	Mean ± SD	66.83 ± 25.08	60.46 ± 22.5	0.548 ** d = 0.280
	95% C.I.	35.68–97.98	54.64–66.27	
	Median (IQR)	70.83 (41.67–90)	62.5 (40.42–74.58)	
Score-SP	Mean ± SD	44.44 ± 51.52	38.24 ± 32.23	0.933 r = 0.01
	95% C.I.	0–100	29.91–46.57	
	Median (IQR)	22.2 (0–100)	38.89 (2.78–66.67)	

* Mann–Whitney U Test, ** Student *t*-Test.

**Table 6:** Comparison of analyzed scores according to the existence of fertility preservation procedure.

Fertility Preservation/Score		Absent	Present	*p* *
Score-PF	Mean ± SD	81.6 ± 16.32	87.88 ± 10.25	0.334 r = 0.12
	95% C.I.	77.15–86.06	81–94.76	
	Median (IQR)	86.7 (73.3–93.3)	86.7 (80–100)	
Score-RF	Mean ± SD	78.1 ± 26.26	89.4 ± 15.4	0.193 r = 0.161
	95% C.I.	70.92–85.26	79.04–99.74	
	Median (IQR)	83.3 (66.7–100)	100 (66.7–100)	
Score-EF	Mean ± SD	61.88 ± 26.82	61.36 ± 27.45	0.860 r = 0.021
	95% C.I.	54.56–69.20	42.92–79.81	
	Median (IQR)	66.7 (47.9–83.3)	66.7 (33.3–83.3)	
Score-CF	Mean ± SD	65.74 ± 27.55	63.64 ± 25.62	0.675 r = 0.052
	95% C.I.	58.22–73.26	46.52–80.85	
	Median (IQR)	66.7 (50–83.3)	66.7 (33.3–83.3)	
Score-SF	Mean ± SD	65.12 ± 24.92	80.3 ± 16.36	0.063 r = 0.23
	95% C.I.	58.32–71.93	69.31–91.29	
	Median (IQR)	66.7 (62.5–83.3)	66.7 (66.7–100)	
Score-Fatigue	Mean ± SD	44.24 ± 27.34	46.46 ± 28.46	0.853 r = 0.023
	95% C.I.	36.78–51.70	27.34–65.59	
	Median (IQR)	38.9 (22.2–66.7)	33.3 (22.2–77.7)	
Score-Nausea	Mean ± SD	14.51 ± 17.74	6.06 ± 15.4	0.066 r = 0.228
	95% C.I.	9.66–19.35	0–16.41	
	Median (IQR)	16.67 (0–16.67)	0 (0–0)	
Score-Pain	Mean ± SD	36.11 ± 26.25	28.8 ± 27.98	0.325 r = 0.122
	95% C.I.	28.95–43.28	10–47.58	
	Median (IQR)	33.3 (16.7–50)	16.7 (0–50)	
Score-Dyspnea	Mean ± SD	17.9 ± 26.47	18.1 ± 27.34	0.992 r = 0.001
	95% C.I.	10.68–25.13	0–36.55	
	Median (IQR)	0 (0–33.3)	0 (0–33.3)	
Score-Insomnia	Mean ± SD	43.21 ± 30.8	45.45 ± 37.33	0.869 r = 0.020
	95% C.I.	34.80–51.62	20.37–70.54	
	Median (IQR)	33.3 (33.3–66.7)	33.3 (0–66.7)	
Score-Appetite	Mean ± SD	15.43 ± 26.47	21.21 ± 37.33	0.992 r = 0.001
	95% C.I.	8.21–22.66	0–46.3	
	Median (IQR)	0 (0–33.3)	0 (0–66.7)	
Score-Constipation	Mean ± SD	22.2 ± 28.22	24.24 ± 26.2	0.686 r = 0.05
	95% C.I.	14.52–29.93	6.64–41.85	
	Median (IQR)	0 (0–33.3)	33.3 (0–33.3)	
Score-Diarrhea	Mean ± SD	17.9 ± 28.01	27.27 ± 35.95	0.437 r = 0.096
	95% C.I.	10.26–25.55	3.12–51.43	
	Median (IQR)	0 (0–33.3)	0 (0–66.7)	
Score-FD	Mean ± SD	32.72 ± 31.38	24.24 ± 33.63	0.349 r = 0.116
	95% C.I.	24.15–41.28	1.65–46.84	
	Median (IQR)	33.3 (0–41.67)	0 (0–66.7)	
Score-GH	Mean ± SD	69.14 ± 15.83	64.4 ± 23.59	0.725 r = 0.043
	95% C.I.	64.81–73.46	48.54–80.25	
	Median (IQR)	66.7 (64.58–83.3)	66.7 (66.7–83.3)	
Score-SA	Mean ± SD	53.7 ± 23.71	33.3 ± 29.81	**0.015** r = 0.3
	95% C.I.	47.23–60.18	13.30–53.36	
	Median (IQR)	66.7 (33.3–66.7)	33.3 (0–33.3)	
Score-DL	Mean ± SD	64.81 ± 32.64	60.61 ± 41.68	0.891 r = 0.017
	95% C.I.	55.9–73.73	32.6–88.61	
	Median (IQR)	66.7 (33.3–100)	66.7 (0–100)	
Score-IN	Mean ± SD	30.25 ± 26.9	24.24 ± 30.15	0.467 r = 0.09
	95% C.I.	22.9–37.6	4–44.5	
	Median (IQR)	33.3 (0–41.67)	0 (0–66.7)	
Score-FA	Mean ± SD	53.09 ± 33.34	39.39 ± 38.92	0.231 r = 0.148
	95% C.I.	43.98–62.2	13.24–65.54	
	Median (IQR)	66.7 (33.3–66.7)	33.3 (0–66.7)	
Score-TR	Mean ± SD	60.5 ± 33.7	48.48 ± 40.45	0.376 r = 0.109
	95% C.I.	51.3–69.7	21.3–75.66	
	Median (IQR)	66.7 (33.3–100)	66.7 (0–66.7)	
Score-CO	Mean ± SD	80.86 ± 23.88	84.85 ± 22.91	0.587 r = 0.067
	95% C.I.	74.35–87.38	69.45–100	
	Median (IQR)	100 (66.7–100)	100 (66.7–100)	
Score-PA	Mean ± SD	41.36 ± 34.22	51.52 ± 37.6	0.364 r = 0.112
	95% C.I.	32.02–50.7	26.25–76.78	
	Median (IQR)	33.3 (0–66.7)	66.7 (0–66.7)	
Score-BI	Mean ± SD	50.62 ± 33.48	48.48 ± 40.45	0.834 r = 0.103
	95% C.I.	41.48–59.76	21.31–75.66	
	Median (IQR)	33.33 (33.3–66.7)	33.3 (0–100)	
Score-VD	Mean ± SD	52.03 ± 22.42	45.83 ± 30.53	0.801 r = 0.04
	95% C.I.	44.96–59.11	20.3–71.36	
	Median (IQR)	66.7 (33.3–66.7)	66.7 (8.33–66.7)	
Score-SS	Mean ± SD	61.02 ± 20	60.61 ± 33.71	0.752 r = 0.039
	95% C.I.	55.56–66.48	37.96–83.26	
	Median (IQR)	64.58 (39.3–73.7)	62.5 (45.8–100)	
Score-SP	Mean ± SD	38.68 ± 33.07	38.9 ± 37.6	0.972 r = 0.004
	95% C.I.	29.66–47.71	13.63–64.15	
	Median (IQR)	38.9 (0–66.7)	33.3 (0–77.7)	

* Mann-Whitney U Test.

## Data Availability

The data presented in this study are available upon reasonable request from the corresponding author. Due to the inclusion of sensitive patient-related information, the data are not publicly available in order to protect patient privacy and confidentiality.
